# Association Among Local Hemodynamic Parameters Derived From CT Angiography and Their Comparable Implications in Development of Acute Coronary Syndrome

**DOI:** 10.3389/fcvm.2021.713835

**Published:** 2021-09-13

**Authors:** Seokhun Yang, Gilwoo Choi, Jinlong Zhang, Joo Myung Lee, Doyeon Hwang, Joon-Hyung Doh, Chang-Wook Nam, Eun-Seok Shin, Young-Seok Cho, Su-Yeon Choi, Eun Ju Chun, Bjarne L. Nørgaard, Koen Nieman, Hiromasa Otake, Martin Penicka, Bernard De Bruyne, Takashi Kubo, Takashi Akasaka, Charles A. Taylor, Bon-Kwon Koo

**Affiliations:** ^1^Department of Internal Medicine and Cardiovascular Center, Seoul National University, Seoul, South Korea; ^2^HeartFlow Inc., Redwood City, CA, United States; ^3^Department of Cardiology, The Second Affiliated Hospital, School of Medicine, Zhejiang University, Hangzhou, China; ^4^Department of Internal Medicine and Cardiovascular Center, Samsung Medical Center, Sungkyunkwan University, Seoul, South Korea; ^5^Department of Medicine, Inje University Ilsan Paik Hospital, Goyang, South Korea; ^6^Department of Medicine, Dongsan Medical Center, Keimyung University, Daegu, South Korea; ^7^Department of Cardiology, Ulsan Hospital, Ulsan, South Korea; ^8^Cardiovascular Center, Sejong General Hospital, Incheon, South Korea; ^9^Department of Medicine, Healthcare System Gangnam Center, Seoul National University, Seoul, South Korea; ^10^Department of Radiology, Seoul National University Bundang Hospital, Seongnam, South Korea; ^11^Department of Cardiology, Aarhus University Hospital, Aarhus, Denmark; ^12^School of Medicine, Cardiovascular Institute, Stanford University, Stanford, CA, United States; ^13^Division of Cardiovascular and Respiratory Medicine, Department of Internal Medicine, Graduate School of Medicine, Kobe University, Kobe, Japan; ^14^Cardiovascular Center Aalst, OLV-Clinic, Aalst, Belgium; ^15^Department of Cardiovascular Medicine, Wakayama Medical University, Wakayama, Japan; ^16^Department of Bioengineering, Stanford University, Stanford, CA, United States; ^17^Institute on Aging, Seoul National University, Seoul, South Korea

**Keywords:** acute coronary syndrome, atherosclerosis, local hemodynamic parameters, coronary artery disease, coronary CT angiography

## Abstract

**Background:** Association among local hemodynamic parameters and their implications in development of acute coronary syndrome (ACS) have not been fully investigated.

**Methods:** A total of 216 lesions in ACS patients undergoing coronary CT angiography (CCTA) before 1–24 months from ACS event were analyzed. High-risk plaque on CCTA was defined as a plaque with ≥2 of low-attenuation plaque, positive remodeling, spotty calcification, and napkin-ring sign. With the use of computational fluid dynamics analysis, fractional flow reserve (FFR) derived from CCTA (FFR_CT_) and local hemodynamic parameters including wall shear stress (WSS), axial plaque stress (APS), pressure gradient (PG) across the lesion, and delta FFR_CT_ across the lesion (ΔFFR_CT_) were obtained. The association among local hemodynamics and their discrimination ability for culprit lesions from non-culprit lesions were compared.

**Results:** A total of 66 culprit lesions for later ACS and 150 non-culprit lesions were identified. WSS, APS, PG, and ΔFFR_CT_ were strongly correlated with each other (all *p* < 0.001). This association was persistent in all lesion subtypes according to a vessel, lesion location, anatomical severity, high-risk plaque, or FFR_CT_ ≤ 0.80. In discrimination of culprit lesions causing ACS from non-culprit lesions, WSS, PG, APS, and ΔFFR_CT_ were independent predictors after adjustment for lesion characteristics, high-risk plaque, and FFR_CT_ ≤ 0.80; and all local hemodynamic parameters significantly improved the predictive value for culprit lesions of high-risk plaque and FFR_CT_ ≤ 0.80 (all *p* < 0.05). The risk prediction model for culprit lesions with FFR_CT_ ≤ 0.80, high-risk plaque, and ΔFFR_CT_ had a similar or superior discrimination ability to that with FFR_CT_ ≤ 0.80, high-risk plaque, and WSS, APS, or PG; and the addition of WSS, APS, or PG into ΔFFR_CT_ did not improve the model performance.

**Conclusions:** Local hemodynamic indices were significantly intercorrelated, and all indices similarly provided additive and independent predictive values for ACS risk over high-risk plaque and impaired FFR_CT_.

## Introduction

Acute coronary syndrome (ACS) is one of the leading causes of death in most countries (1), and predicting ACS risk prior to fatal events has been a major challenge in patients with coronary artery disease. Pathological studies demonstrated the vulnerable plaque features closely related to ACS (2), and identification of high-risk plaque features on coronary imaging provided better risk prediction for future events (3, 4). However, coronary anatomy or plaque morphology-based evaluation has shown a low positive predictive value in predicting ACS (5). Coronary physiological assessment such as fractional flow reserve (FFR), a guiding tool for appropriate revascularization in a current guideline (6), has an excellent negative predictive value for ACS, but its low likelihood ratio of ACS has also been reported in major randomized controlled trials (7, 8).

Unfavorable local hemodynamic environment has a critical role in ACS development (9). Plaque rupture commonly occurs when external forces acting on a plaque exceed plaque strength, and these forces can be estimated by the pressure drop across a lesion (10). Wall shear stress (WSS), a tiny tangential force, is known as a proinflammatory stimulus leading to plaque formation, progression, and destabilization prone to rupture events (11). Therefore, it has been speculated that identification of local hemodynamic parameters displayed better prediction of plaque rupture risk (12). Nonetheless, their clinical utilization has still been limited in daily practice since it requires additional resources and is a time-consuming process (13, 14). Moreover, whether the assessment of all diverse local hemodynamic indices provides incremental value has not been fully understood. In this regard, we performed this study to investigate the relationship among various local hemodynamic parameters and their comparability in prediction of ACS risk.

## Methods

### Study Participants

This study is a substudy of the EMERALD (Exploring the Mechanism of Plaque Rupture in Acute Coronary Syndrome Using Coronary CT Angiography and Computational Fluid Dynamic) study (12). Patients with ACS defined as acute myocardial infarction or unstable angina with evidence of plaque rupture at invasive coronary imaging at the time of ACS and who underwent coronary CT angiography (CCTA) from 1 month to 2 years before ACS event were included. All angiograms were reviewed at a core laboratory, Seoul National University Hospital; and the culprit lesions were selected in a blinded fashion. Those with ACS due to in-stent restenosis, secondary myocardial infarction due to other general medical conditions, previous history of coronary artery bypass graft surgery, or unanalyzable CCTA for computational fluid dynamics (CFD) analysis at a core laboratory were excluded. The study protocol was approved by the institutional review board of each site. The study was conducted in accordance with the Declaration of Helsinki (ClinicalTrials.gov Identifier: NCT02374775).

### Plaque Analysis on Coronary CT Angiography

CCTA images were analyzed at a core laboratory (Seoul National University Bundang Hospital) by an independent observer in a blinded fashion. All lesions with percent diameter stenosis >30% were analyzed. Lesion starting and ending locations were visually determined on the basis of lumen geometry by an independent reviewer. The presence of low-attenuation plaque (LAP) was defined as a plaque with an average density of ≤ 30 Hounsfield units [HU]) (15), which was obtained by the mean value of HU randomly selected ≥5 points in the lesion. Positive remodeling (PR) was defined as a remodeling index ≥1.1 (15). The remodeling index was defined as the vessel diameter at the maximal stenotic site divided by the reference diameter. Spotty calcification was a lesion with averaged density >130 HU and diameter <3 mm in any direction, and napkin-ring sign was ring-like attenuation pattern with peripheral high and central lower-attenuation portion. High-risk plaque was defined as a plaque with ≥2 of LAP, PR, spotty calcification, and napkin-ring sign.

### Hemodynamic Parameters From Coronary CT Angiography Images

Hemodynamic parameters were obtained by CFD analyses on CCTA in a blinded fashion at an independent core laboratory (Heart Flow, Inc.) (16, 17). CFD analyses were performed by the same process performed during FFR_CT_ computation. In brief, the individual anatomic model of coronary arteries was reconstructed from CCTA images, and segmentation of lumen boundary was performed. Blood flow and pressure in the coronary trees were predicted using the CFD technique by solving the Navier–Stokes equations with the assumptions of a rigid wall and a Newtonian fluid in a patient-specific coronary geometry (16). Myocardial mass, vessel sizes at each outlet, and the microvascular response to adenosine were used for defining the boundary conditions. Following the principles that coronary supply meets myocardial demand of each patient at rest, and microvascular resistance at rest has an inverse relationship linearly proportional to the size of the vessel (18), and microcirculatory reaction to maximal hyperemia in patients with the normal coronary flow is predictable (19), total coronary flow at rest and the total baseline resistance were computed. The total baseline resistance was distributed to coronary trees on the basis of vessel caliber and was reduced according to the effect of adenosine on the microvasculature in hyperemic conditions. The inflow condition was determined by the patient-specific myocardial mass and functional relationships between flow and mass based on the allometric scaling law. The simulations were conducted under the steady flow assumption, and all hemodynamic parameters were calculated in hyperemic conditions. We obtained FFR_CT_, change in FFR_CT_ across the lesion (ΔFFR_CT_), WSS, axial plaque stress (APS), and pressure gradient (PG) across the lesion. Over the entire coronary tress, the hemodynamic quantities can be achieved by FFR_CT_ tracing. Whole coronary artery tree was sliced by a unit of a thin strip with 2-mm thickness with 0.5-mm intervals between strips. Then, the averaged values of hemodynamic parameters of every strip could be obtained, and hemodynamic properties of the whole coronary artery were identified. The definitions of hemodynamic parameters were as follows. FFR_CT_ was defined as (mean pressure in downstream coronary vessels/mean pressure of the aorta) under simulated hyperemic conditions at the distal part of a vessel. WSS was defined as tangential stress resulting from the friction between blood flow and the surface of the vessel wall. PG was defined as the difference between the proximal and distal pressure divided by lesion length. Given that the pressure drop across the lesion mainly occurs along the axial direction, the axial component of the traction was separately defined as APS, a measure for the main driving force along the vessel length (10). To obtain the net resultant forces acting on the plaque, we used hemodynamic parameters averaged over the surface of each lesion in the current analysis. ΔFFR_CT_ was calculated as the difference between the FFR_CT_ value at a lesion start point (i.e., proximal FFR_CT_) and the FFR_CT_ value at a lesion endpoint (i.e., distal FFR_CT_). Sampling points for ΔFFR_CT_ were equal to those for PG. As a sensitivity analysis, averaged WSS was divided into proximal WSS and distal WSS based on the point of minimum lumen area; and their association with other hemodynamic parameters, prognostic implications, and the additive value for ΔFFR_CT_ was analyzed. Optimal cutoff based on receiver operating characteristic curve analysis in discrimination of culprit lesions from non-culprit lesions was used to define high WSS (≥154.7 dyn/cm^2^), high APS (≥1,606.6 dyn/cm^2^), high ΔFFR_CT_ (≥0.06), and high PG (≥5.8 mmHg/cm). The optimal cutoff providing the maximal value of the sum of sensitivity and specificity was chosen, and the same cutoff was used in the original EMERALD study (12).

### Statistical Analysis

All analyses were performed using R language version 3.6.2 (R Foundation for Statistical Computing, Vienna, Austria). Continuous variables were expressed as means with standard deviations. Categorical variables were shown as numbers (percentages). Two or more groups were compared using Student's *t*-test test or ANOVA test for continuous variables and chi-square test for categorical variables, as appropriate. Pearson's correlation coefficient was used to assess the linear association among hemodynamic parameters and was estimated according to the lesion subtypes stratified by a vessel, lesion location, % diameter stenosis, high-risk plaque, FFR_CT_, and the number of lesions in a vessel. Global chi-square estimates were used to evaluate the additive predictive value of local hemodynamics over the presence of high-risk plaque and FFR_CT_ ≤ 0.80. The cumulative event rates were assessed by the Kaplan–Meier censoring estimates. Cox proportional hazard regression was used to estimate the hazard ratio (HR) and the corresponding 95% confidence interval (CI). For adjustment of intra-patient variability in the same patient, the marginal Cox model was used. In the multivariate analysis, lesion characteristics significantly different between culprit and non-culprit lesions (i.e., vessel location, % diameter stenosis, and lesion length), FFR_CT_ ≤ 0.80, high-risk plaque, and each local hemodynamic parameter were included in the Cox model. The discrimination ability for culprit lesions from non-culprit lesions was compared to assess comparability among local hemodynamics using the area under the receiver operating characteristic curve (AUC) based on logistic regression. A generalized estimating equation was used for the adjustment of intra-patient variability. All statistical tests were two-tailed, and a *p*-value < 0.05 was considered statistically significant.

## Results

### Baseline Characteristics of Patients and Lesions

**Figure 1:**
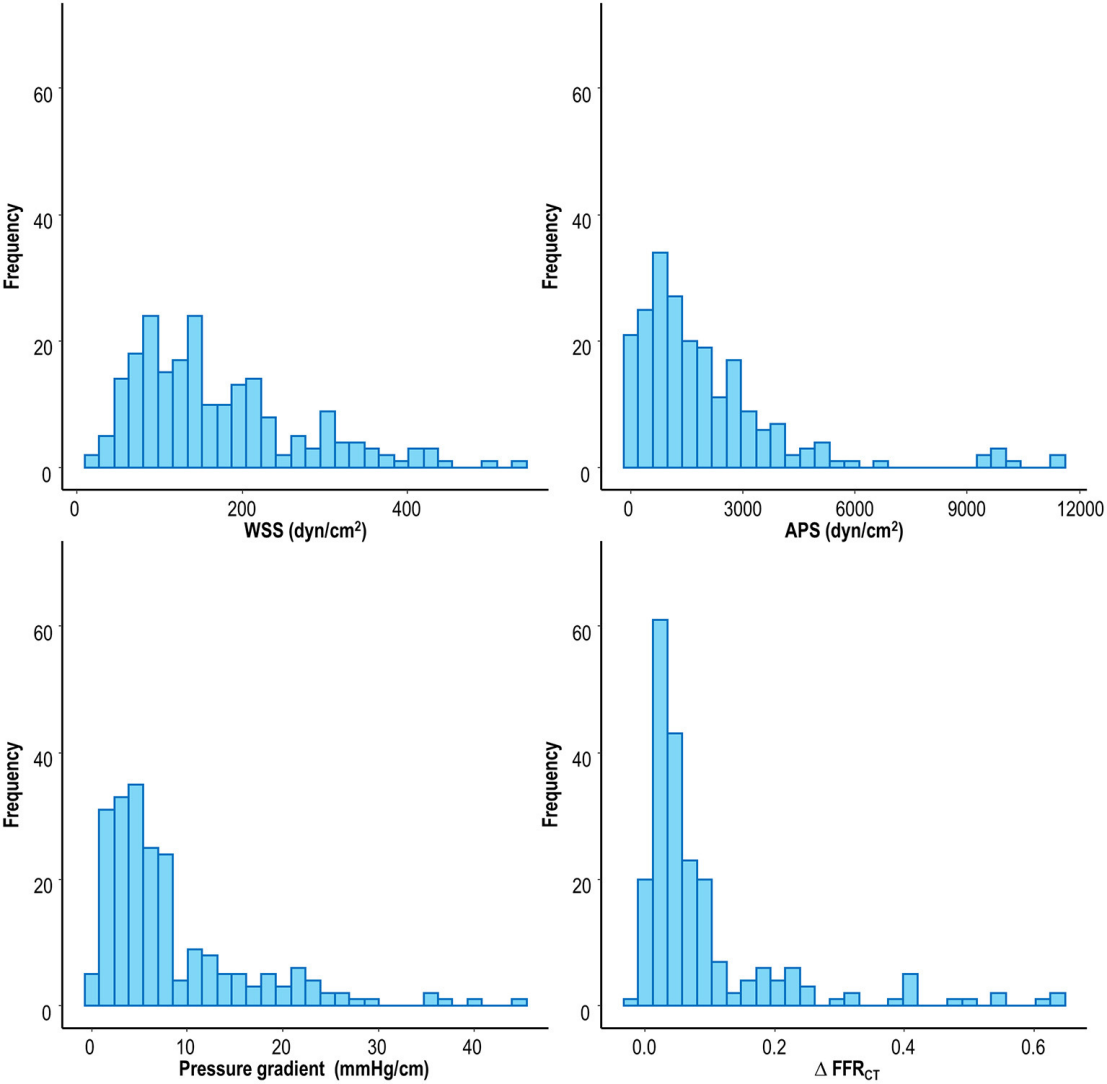
Distribution of local hemodynamic parameters. APS, axial plaque stress; FFR_CT_, coronary computed tomographic angiography-derived fractional flow reserve; WSS, wall shear stress.

Among 72 patients with ACS, the mean age of the study population was 69.9 ± 12.7 years, and 54% were male; and the proportion of patients with diabetes mellitus, hypertension, and hypercholesterolemia was 51.4, 63.9, and 48.6%, respectively. Current smoker was 30.6%, and 6.9% had a previous history of myocardial infarction. The median ejection fraction was 58.6 (44.5–63.3%). The median interval from CCTA to ACS events was 338.0 (161.5–535.0) days, and ACS events were comprised by 93.1% of myocardial infarction and 6.9% of unstable angina. Of these patients, a total of 216 lesions were identified on CCTA taken prior to ACS, including 66 culprit lesions and 150 non-culprit lesions. Relative to non-culprit lesions, culprit lesions showed a higher % diameter stenosis (43.1 ± 15.0% vs. 55.5 ± 15.4%, *p* < 0.001), and lesion length (12.1 ± 7.4 mm vs. 15.8 ± 8.4 mm, *p* = 0.002). The proportion of located lesions on the left anterior descending artery, left circumflex artery, and right coronary artery was 59.1, 13.6, and 27.3%, respectively in culprit lesions; and 32.0, 26.0, and 42.0%, respectively, in non-culprit lesions (*p* < 0.001). Time between CCTA and ACS events was 271.5 [116.0–522.0] days in culprit lesions and 338.5 [164.0–535.0] days in non-culprit lesions (*p* = 0.149). The distributions of local hemodynamic parameters are presented in Figure 1. The value of local hemodynamic parameters by lesion characteristics is shown in Table 1; and the trends according to lesion characteristics are similar among WSS, APS, PG, and ΔFFR_CT_.

**Table 1:** Local hemodynamics according to lesion characteristics.

	**WSS (dyn/cm^**2**^)**	***P*-value**	**APS (dyn/cm^**2**^)**	***P*-value**	**PG (mmHg/cm)**	***P*-value**	**ΔFFR_**CT**_**	***P*-value**
**Total**	168.8 ± 102.1	–	1,994.8 ± 2,095.5	–	8.3 ± 7.9	–	0.09 ± 0.12	–
**Vessel location**		<0.001		0.017		<0.001		0.016
LAD (*n* = 87)	208.8 ± 105.8		2,341.9 ± 2,266.9		10.9 ± 8.1		0.12 ± 0.13	
LCX (*n* = 48)	158.6 ± 99.6		2,083.2 ± 2,056.6		8.2 ± 9.4		0.08 ± 0.11	
RCA (*n* = 81)	131.9 ± 83.6		1,569.6 ± 1,863.7		5.6 ± 5.6		0.07 ± 0.11	
**Lesion location**		<0.001		0.033		<0.001		0.006
Proximal (*n* = 98)	201.9 ± 113.3		2,453.6 ± 2,418.2		10.5 ± 9.2		0.12 ± 0.14	
Middle (*n* = 81)	150.5 ± 90.0		1,482.6 ± 1,597.1		7.1 ± 6.7		0.08 ± 0.01	
Distal (*n* = 37)	121.2 ± 59.7		1,900.8 ± 1,904.5		5.3 ± 4.7		0.06 ± 0.08	
**% Diameter stenosis**		<0.001		0.001		<0.001		<0.001
≥50% (*n* = 84)	206.2 ± 121.7		2,656.8 ± 2,598.5		11.9 ± 10.1		0.15 ± 0.17	
<50% (n = 132)	145.0 ± 79.1		1,573.5 ± 1,571.2		6.0 ± 4.9		0.05 ± 0.05	
**High-risk plaque**		0.003		0.042		0.002		<0.001
Yes (*n* = 60)	201.4 ± 102.4		2,547.2 ± 2,641.4		11.3 ± 9.2		0.16 ± 0.18	
No (*n* = 156)	156.3 ± 99.5		1,782.3 ± 1,808.7		7.2 ± 7.0		0.07 ± 0.08	
**FFR**_**CT**_		<0.001		0.097		<0.001		<0.001
≤ 0.80 (*n* = 66)	227.7 ± 127.1		2,351.8 ± 2,242.8		14.1 ± 10.5		0.19 ± 0.18	
>0.80 (*n* = 150)	142.9 ± 76.1		1,837.7 ± 2,015.0		5.8 ± 4.5		0.05 ± 0.04	
**Number of lesions in a vessel**		0.245		0.009		0.101		0.200
1 (*n* = 86)	158.9 ± 95.1		1,577.8 ± 1,482.9		7.3 ± 6.4		0.08 ± 0.09	
≥ 2 (*n* = 130)	175.4 ± 106.4		2,270.6 ± 2,382.6		9.0 ± 8.7		0.10 ± 0.14	

*High-risk plaque was defined as a plaque with ≥2 of low-attenuation plaque, positive remodeling, spotty calcification, and napkin-ring sign*.

*APS, axial plaque stress; FFR_CT_, coronary computed tomographic angiography-derived fractional flow reserve; LAD, left anterior descending artery; LAP, low-attenuation plaque; LCX, left circumflex artery; PG, pressure gradient; PR, positive remodeling; RCA, right coronary artery; WSS, wall shear stress*.

### Relationship Among Local Hemodynamic Parameters

**Figure 2:**
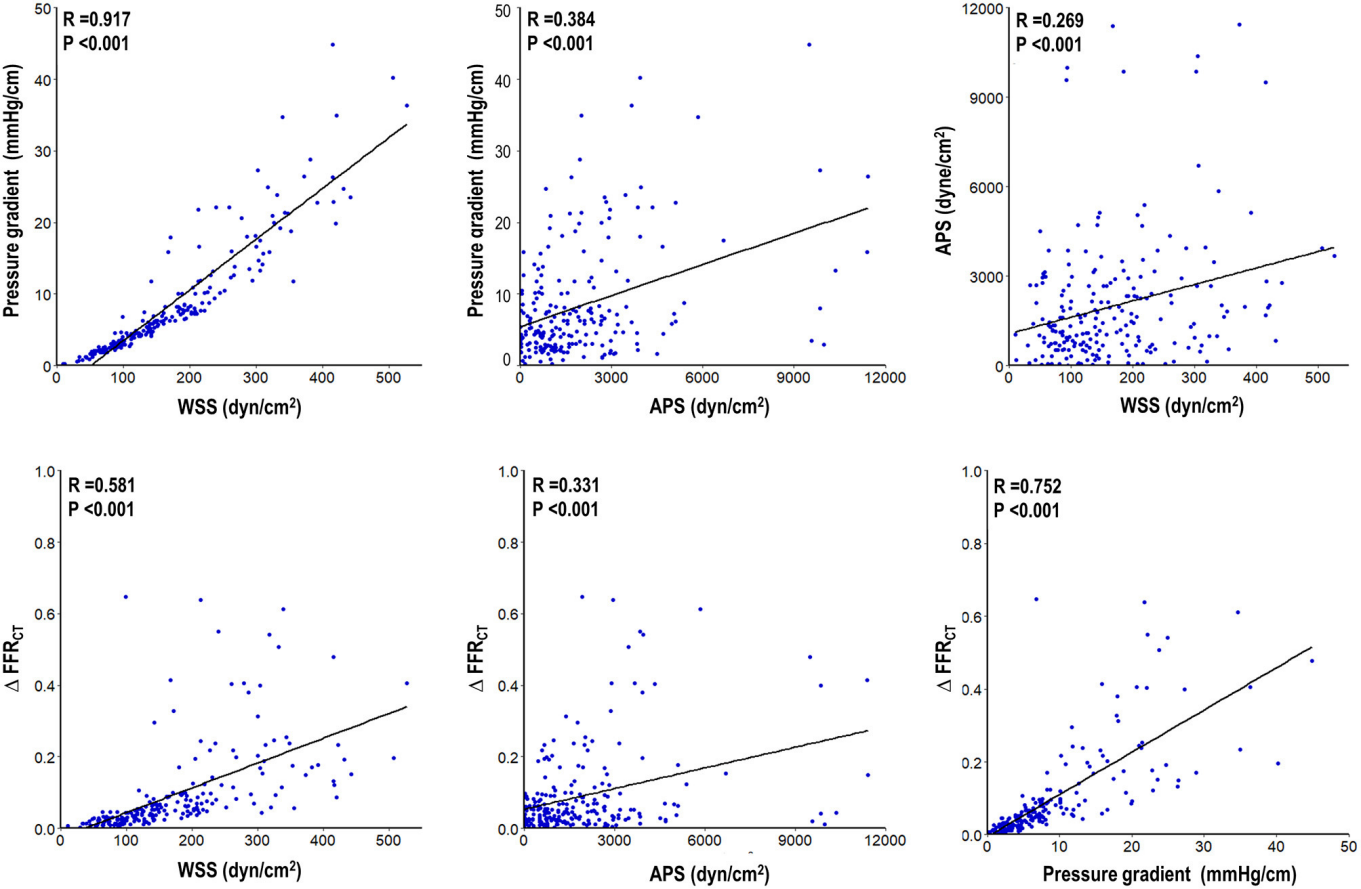
Association among local hemodynamic parameters. Correlation among WSS, APS, PG, and ΔFFR_CT_ is presented. All local hemodynamic parameters were significantly correlated with each other. APS, axial plaque stress; FFR_CT_, coronary computed tomographic angiography-derived fractional flow reserve; WSS, wall shear stress.

Figure 2 describes the association among hemodynamic parameters. WSS, APS, and PG had a significant correlation among each other (*r* = 0.917, *p* < 0.001 for WSS and PG; *r* = 0.384, *p* < 0.001 for APS and PG; and *r* = 0.269, *p* < 0.001 for WSS and APS). ΔFFR_CT_ was significantly correlated with WSS, APS, and PG (*r* = 0.581, *p* < 0.001 for WSS and ΔFFR_CT_; *r* = 0.331, *p* < 0.001 for APS and ΔFFR_CT_; and *r* = 0.752, *p* < 0.001 for PG and ΔFFR_CT_) (Figure 2). In regression of WSS, APS, or PG, the correlation coefficient of FFR_CT_ was lower than that of ΔFFR_CT_ with WSS, APS, or PG (*r* = −0.349, *p* < 0.001 for WSS and FFR_CT_; *r* = −0.131, *p* < 0.001 for APS and FFR_CT_; and *r* = −0.526, *p* < 0.001 for PG and FFR_CT_) (Supplementary Figure 1). In various lesion subtypes stratified by a vessel, lesion location, % diameter stenosis, high-risk plaque, FFR_CT_, and number of lesions in a vessel, local hemodynamic indices consistently correlated with each other (Table 2), although the correlation of APS with other local hemodynamic parameters becomes weak in lesions with <50% diameter stenosis. FFR_CT_ showed a lower correlation coefficient with WSS, APS, or PG than that of ΔFFR_CT_ with WSS, APS, or PG in overall lesion types (Supplementary Table 1). In particular, FFR_CT_ did not correlate with local hemodynamics in lesions with FFR_CT_ ≤ 0.80, and the correlation of FFR_CT_ with local hemodynamics decreased in vessels with multiple lesions (Supplementary Table 1).

**Table 2:** Correlation among local hemodynamic parameters in various lesion subtypes.

**Subgroups**	**Correlation between WSS and PG (coefficient, r)**	***P*-value**	**Correlation between APS and PG (coefficient, r)**	***P*-value**	**Correlation between WSS and APS (coefficient, r)**	***P*-value**	**Correlation between ΔFFR_**CT**_ and WSS (coefficient, r)**	***P*-value**	**Correlation between ΔFFR_**CT**_ and APS (coefficient, r)**	***P*-value**	**Correlation between ΔFFR_**CT**_ and PG (coefficient, r)**	***P*-value**
**Vessel location**												
LAD (*n* = 81)	0.906	<0.001	0.287	0.007	0.159	0.142	0.541	<0.001	0.290	<0.001	0.762	<0.001
LCX (*n* = 48)	0.915	<0.001	0.596	<0.001	0.400	0.005	0.653	<0.001	0.621	<0.001	0.811	<0.001
RCA (*n* = 81)	0.941	<0.001	0.245	0.028	0.225	0.044	0.543	<0.001	0.152	0.177	0.703	<0.001
**Lesion location**												
Proximal (*n* = 98)	0.915	<0.001	0.299	0.003	0.173	0.088	0.548	<0.001	0.186	0.067	0.712	<0.001
Mid (*n* = 81)	0.910	<0.001	0.423	<0.001	0.306	0.006	0.566	<0.001	0.532	<0.001	0.777	<0.001
Distal (*n* = 37)	0.890	<0.001	0.638	<0.001	0.444	0.006	0.637	<0.001	0.579	<0.001	0.866	<0.001
**% diameter stenosis**												
≥50% (*n* = 84)	0.905	<0.001	0.453	<0.001	0.319	0.003	0.493	<0.001	0.377	<0.001	0.699	<0.001
<50% (*n* = 132)	0.948	<0.001	0.052	0.558	0.035	0.688	0.735	<0.001	−0.072	0.412	0.802	<0.001
**High-risk plaque**												
Yes (*n* = 60)	0.875	<0.001	0.295	0.022	0.111	0.398	0.492	<0.001	0.293	0.023	0.738	<0.001
No (*n* = 156)	0.943	<0.001	0.409	<0.001	0.326	<0.001	0.687	<0.001	0.316	<0.001	0.781	<0.001
**FFR**_**CT**_												
≤ 0.80 (*n* = 66)	0.903	<0.001	0.559	<0.001	0.352	0.004	0.458	<0.001	0.544	<0.001	0.662	<0.001
>0.80 (*n* = 150)	0.941	<0.001	0.232	0.004	0.168	0.040	0.788	<0.001	0.073	0.374	0.819	<0.001
**Number of lesions in a vessel**												
1 (*n* = 86)	0.961	<0.001	0.128	0.242	0.058	0.596	0.735	<0.001	0.184	0.089	0.831	<0.001
≥2 (*n* = 130)	0.903	<0.001	0.449	<0.001	0.338	<0.001	0.526	<0.001	0.361	<0.001	0.725	<0.001

*The definition of high-risk plaque was the same as in Table 1*.

*APS, axial plaque stress; FFR_CT_, coronary computed tomographic angiography-derived fractional flow reserve; LAD, left anterior descending artery; LAP, low-attenuation plaque; LCX, left circumflex artery; PG, pressure gradient; PR, positive remodeling; RCA, right coronary artery; WSS, wall shear stress*.

### Comparable Implications of Each Local Hemodynamic Parameter in Prediction of Acute Coronary Syndrome

**Figure 3:**
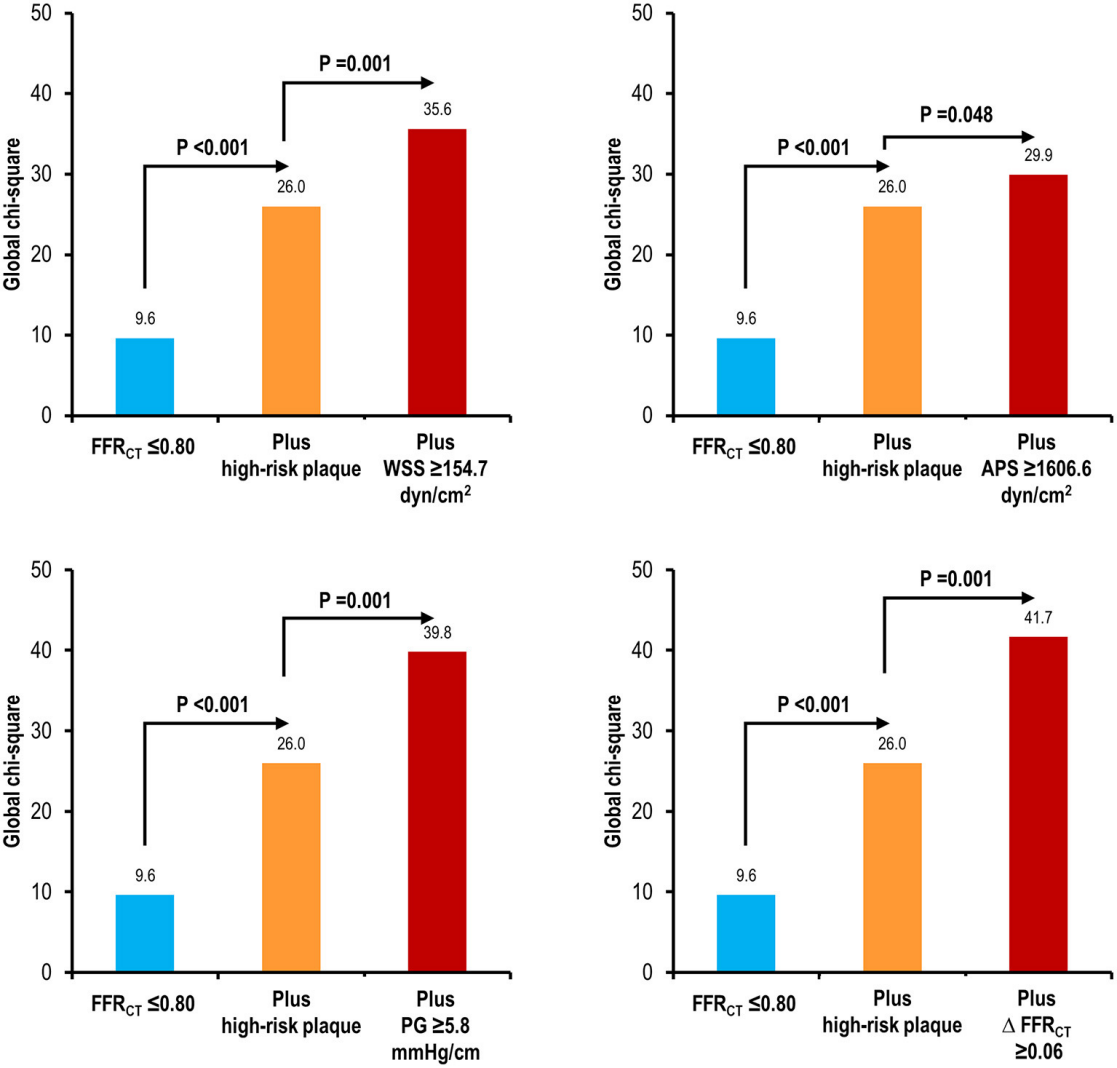
Incremental predictive value of high WSS, high APS, high PG, or high ΔFFR_CT_ over high-risk plaque and FFR_CT_. The predictability for culprit lesions causing ACS is compared with the model with FFR_CT_ ≤ 0.80; the model with FFR_CT_ ≤ 0.80 and high-risk plaque; and the model with FFR_CT_ ≤ 0.80, high-risk plaque, and local hemodynamic parameters. High WSS, high APS, high PG, or high ΔFFR_CT_ similarly improved the predictability for culprit lesions causing ACS of high-risk plaque and FFR_CT_ ≤ 0.80. High-risk plaque was defined as a plaque with ≥2 of low-attenuation plaque, positive remodeling, spotty calcification, and napkin-ring sign. APS, axial plaque stress; FFR_CT_, coronary computed tomographic angiography-derived fractional flow reserve; PG, pressure gradient; WSS, wall shear stress.

**Figure 4:**
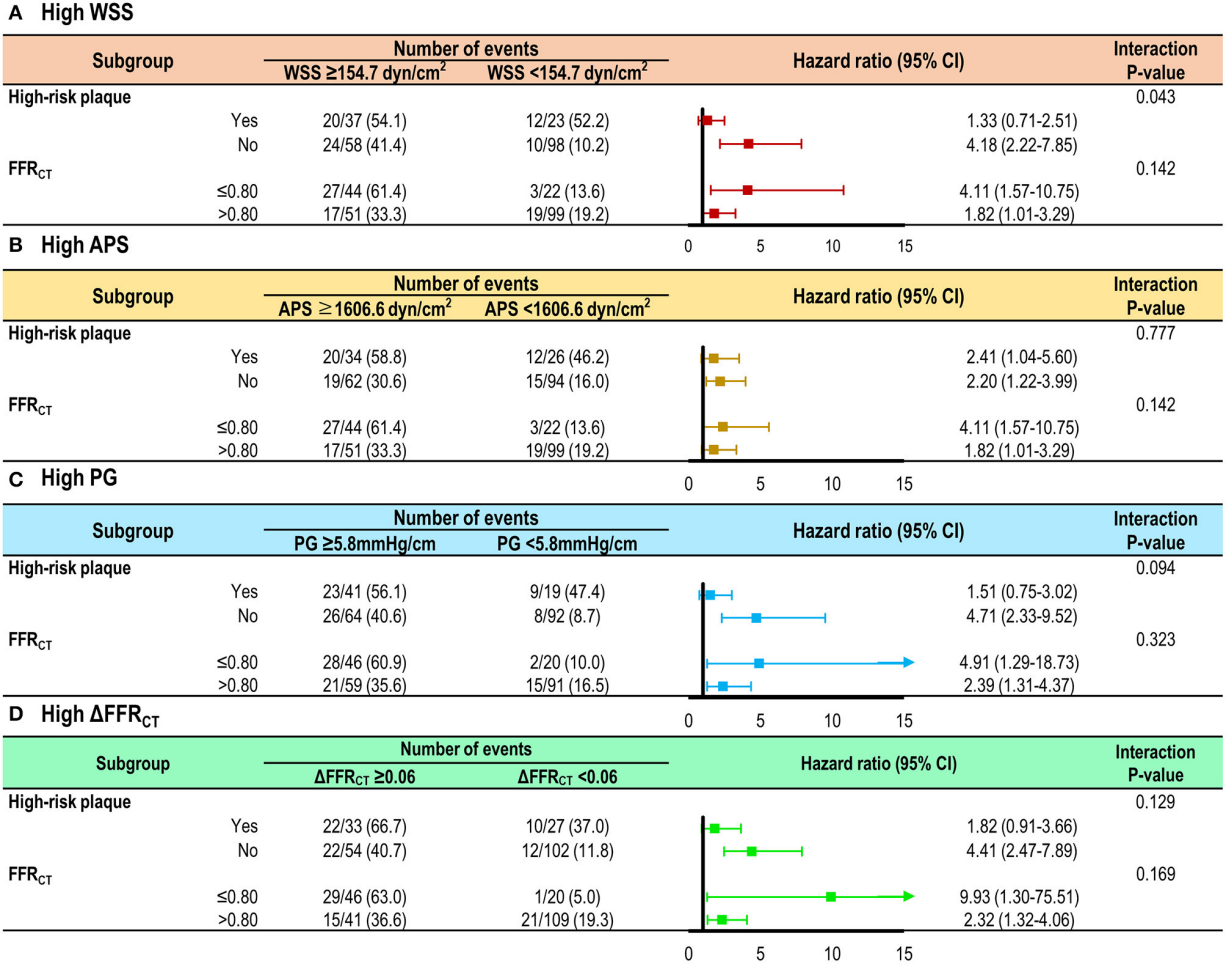
Risk of culprit lesions according to local hemodynamic parameters in the subgroups by high-risk plaque or FFR_CT_. The risk of culprit lesions according to **(A)** high WSS, **(B)** high APS, **(C)** high PG, or **(D)** high ΔFFR_CT_ is shown in lesions with and without high-risk plaque and FFR_CT_ ≤ 0.80. A trend toward an increased risk of culprit lesions was consistently observed in lesions with high WSS, high APS, high PG, or high ΔFFR_CT_, independent of the presence of high-risk plaque and FFR_CT_ ≤ 0.80. The definition of high-risk plaque was the same as in Figure 3. APS, axial plaque stress; FFR_CT_, coronary computed tomographic angiography-derived fractional flow reserve; PG, pressure gradient; WSS, wall shear stress.

In prediction of culprit lesions of ACS, high WSS, high APS, high PG, or high ΔFFR_CT_ was significantly associated with an increased risk after adjustment for vessel location, % diameter stenosis, lesion length, FFR_CT_ ≤ 0.80, and high-risk plaque (adjusted HR 2.02, 95% CI 1.18–3.45, *p* = 0.010 for high WSS; adjusted HR 1.72, 95% CI 1.03–2.88, *p* = 0.038 for high APS; adjusted HR 2.21, 95% CI 1.34–3.67, *p* = 0.002 for high PG; and adjusted HR 2.29, 95% CI 1.35–3.86, *p* = 0.002 for high ΔFFR_CT_) (Table 3). With FFR_CT_ ≤ 0.80 as a baseline model in prediction of culprit lesions, the presence of high-risk plaque had incremental predictability over FFR_CT_ ≤ 0.80; and high WSS, high APS, high PG, or high ΔFFR_CT_ similarly showed an additive predictive value over both FFR_CT_ ≤ 0.80 and high-risk plaque (Figure 3). High WSS, high APS, high PG, or high ΔFFR_CT_ was similar in their ability to discriminate culprit lesions from non-culprit lesions in lesions with and without FFR_CT_ ≤ 0.80 and high-risk plaque (Figure 4). The discrimination ability for culprit lesions of the model with FFR_CT_ ≤ 0.80, high-risk plaque, and ΔFFR_CT_ was similar to that with FFR_CT_ ≤ 0.80, high-risk plaque, and WSS (AUC 0.77 vs. 0.76, *p* = 0.37) or FFR_CT_ ≤ 0.80, high-risk plaque, and PG (0.77 vs. 0.77, *p* = 0.63); or superior to that with FFR_CT_ ≤ 0.80, high-risk plaque, and APS (AUC 0.77 vs. 0.71, *p* = 0.03). The addition of WSS, APS, or PG into ΔFFR_CT_ had no gain in predictive value for culprit lesions (Table 4). Overall results were similar when proximal WSS and distal WSS were separately analyzed in the sensitivity analysis.

**Table 3:** Univariate and multivariate analyses of local hemodynamics in prediction of culprit lesions causing acute coronary syndrome.

	**Unadjusted HR (95% CI)**	***P*-value**	Adjusted HR^*^ (95% CI)	***P*-value**	Adjusted HR^*^ (95% CI)	***P*-value**	Adjusted HR^*^ (95% CI)	***P*-value**	Adjusted HR^*^ (95% CI)	***P*-value**
**Predictors**										
FFR_CT_ ≤ 0.80	2.96 (1.79–4.91)	<0.001	1.51 (0.82–2.78)	0.182	1.78 (0.99–3.23)	0.056	1.47 (0.79–2.76)	0.227	1.27 (0.69–2.34)	0.446
High-risk plaque	3.46 (2.29–5.22)	<0.001	2.08 (1.22–3.56)	0.007	2.24 (1.34–3.73)	0.002	2.10 (1.23–3.58)	0.006	2.03 (1.78–3.51)	0.011
WSS ≥ 154.7 dyn/cm^2^	2.93 (1.88–4.58)	<0.001	2.02 (1.18–3.45)	0.010						
APS ≥ 1,606.6 dyn/cm^2^	2.20 (1.43–3.41)	<0.001			1.72 (1.03–2.88)	0.038				
PG ≥ 5.8 mmHg/cm	3.50 (2.16–5.68)	<0.001					2.21 (1.34–3.67)	0.002		
ΔFFR_CT_ ≥ 0.06	3.70 (2.38–5.76)	<0.001							2.29 (1.35–3.86)	0.002

*The definition of high-risk plaque was the same as in Table 1*.

* *Adjusted for vessel location, % diameter stenosis, lesion length, FFR_CT_ ≤ 0.80, high-risk plaque, and each local hemodynamic parameter*.

*APS, axial plaque stress; CI, confidence interval; FFR_CT_, coronary computed tomographic angiography-derived fractional flow reserve; HR, hazard ratio; PG, pressure gradient; WSS, wall shear stress*.

**Table 4:** ΔFFR_CT_ as a representative marker of local hemodynamic parameters in prediction of culprit lesions causing acute coronary syndrome.

**Model**	**AUC**	***P*-value**
FFR_CT_ ≤ 0.80 + high-risk plaque	0.68	<0.01
FFR_CT_ ≤ 0.80 + high-risk plaque + ΔFFR_CT_ (ref)	0.77	NA
FFR_CT_ ≤ 0.80 + high-risk plaque + WSS	0.76	0.37
FFR_CT_ ≤ 0.80 + high-risk plaque + APS	0.71	0.03
FFR_CT_ ≤ 0.80 + high-risk plaque + PG	0.77	0.63
FFR_CT_ ≤ 0.80 + high-risk plaque + ΔFFR_CT_ + WSS	0.78	0.84
FFR_CT_ ≤ 0.80 + high-risk plaque + ΔFFR_CT_ + APS	0.78	0.58
FFR_CT_ ≤ 0.80 + high-risk plaque + ΔFFR_CT_ + PG	0.78	0.72

*The definition of high-risk plaque was the same as in Table 1*.

*APS, axial plaque stress; AUC, area under curve; FFR_CT_, coronary computed tomographic angiography-derived fractional flow reserve; PG, pressure gradient; WSS, wall shear stress; NA, not applicable*.

## Discussion

The current study investigated the association among local hemodynamic parameters and their role in development of ACS. The main findings were as follows. First, local hemodynamic parameters (i.e., WSS, APS, PG, and ΔFFR_CT_) were significantly correlated with each other. Second, all local hemodynamic indices similarly provided incremental and independent discrimination ability of culprit lesions causing ACS from non-culprit lesions over high-risk plaque and FFR_CT_ ≤ 0.80. Third, ΔFFR_CT_ showed a comparable predictive value for culprit lesions with that of WSS, APS, and PG.

### Components of Local Hemodynamic Environment and Their Association

A large body of evidence has supported the clinical relevance and prognostic value of physiological lesion characteristics in prediction of lesions causing future coronary events (9). It generally consists of endothelial shear stress (ESS) or WSS, a tangential component of force generated by friction between blood flow and vessel wall, which can be sensed by the endothelium leading to a biological process of atherosclerosis (11), and external mechanical force acting on a plaque, which can directly cause plaque rupture when it outpaces plaque strength (10). Although each component of local hemodynamic indices apparently appears to have a different role in a complex process of plaque formation, progression, and rupture events (20), their *in vivo* association and whether they have differential implications in prediction of ACS risk have not been fully understood. It is clinically relevant to investigate their association and compare their prognostic impact on ACS risk, since not all measurements can be obtained in daily practice. In the current study, we employed PG or APS as one of the indicative markers for the external hemodynamic force acting on a plaque, WSS as a local hemodynamic marker of biological signaling on the atherosclerosis process (11), and ΔFFR_CT_ as a clinically applicable marker derived from CFD analyses applied to CCTA taken prior to ACS events.

We demonstrated that WSS, APS, PG, and ΔFFR_CT_ had a significant association with each other (all *p* < 0.001). Moreover, this relationship was consistent, regardless of various lesion subtypes, which indicates that the nature of this firm association among physiological factors was not affected by lesion characteristics. Thus, ΔFFR_CT_ can be a marker of the level of WSS, APS, or PG of a target lesion. This finding may be expected in that each local hemodynamic parameter originates from the common interaction between blood flow, plaque, and vessel wall (20) and is in accordance with previous reports of the strong correlation between WSS and PG at resting (r = 0.969, *p* < 0.001) and hyperemic conditions (*r* = 0.962, *p* < 0.001) in CFD model from CCTA (17), and linear association between APS and PG in obstructive lesions (10). Of note, the degree of correlation of FFR_CT_ with local hemodynamics was lower than that of ΔFFR_CT_, suggesting the importance of lesion-specific hemodynamic assessment than vessel-specific indices to accurately estimate physiological lesion characteristics in clinical practice.

### Additive Value of Local Hemodynamics Relative to High-Risk Plaque and Low FFR in Prediction of Acute Coronary Syndrome

While extensive studies have searched for vulnerable plaque features predictive of future rupture events, major clinical trials have shown that their positive predictive value is far from perfect (3, 21, 22), and the same is true for abnormal coronary physiology (i.e., FFR ≤ 0.80) (7, 8), currently the best indication of revascularization. In the current study, we confirmed the additive and independent predictive value for culprit lesions causing ACS over high-risk plaque and FFR_CT_ ≤ 0.80. High WSS, high APS, high PG, and high ΔFFR_CT_ were independent predictors for culprit lesions after adjustment for high-risk plaque and FFR_CT_ ≤ 0.80, and they all significantly improved the predictability for culprit lesions of the model with high-risk plaque and FFR_CT_ ≤ 0.80 (all *p* < 0.001). Of note, high WSS, high APS, high PG, and high ΔFFR_CT_ were still predictive of culprit lesions irrespective of the presence of high-risk plaque or FFR_CT_ ≤ 0.80. Our finding is in line with the previous report of a strong correlation between the shear stress concentration and plaque rupture site (kappa = 0.79) (23). Although plaque structural stress (PSS) was not estimated in the current study, prior observation of increased PSS or its variability in plaques with rupture (24) partially support our findings of the independent role of local hemodynamics in acute coronary events. *Post-hoc* analysis of FAME II also exhibited that the risk of myocardial infarction can be better predicted by high lesion-level shear stress relative to FFR in medically treated patients with FFR ≤ 0.80 (25). A recent report by Doradla et al. of the precise ability of a peak stress metric in locating the plaque rupture sites also aligns with the current findings (14). Thus, local hemodynamic parameters should be accounted for as one of the main determinants in predicting ACS risk, as they definitely can refine the current risk stratification for ACS with high-risk plaque and low FFR.

### ΔFFR as a Local Hemodynamic Parameter Easily Applicable in Clinical Practice

Various CFD modeling strategies from multimodality imaging have been developed for precise evaluation of the local hemodynamic environment (26). Intravascular ultrasound (IVUS)-derived ESS or PSS has consistently predicted plaque progression, vulnerable plaque formation, and future coronary events (27–32). Recently proposed quantitative coronary angiography-derived ESS showed a correlation with IVUS-derived models (*r* = 0.588, *p* < 0.001) (33) and was an independent predictor of major adverse cardiovascular events at 5-year follow-up (13), suggesting a possibility of real-time assessment of local hemodynamic parameters in daily practice. A novel approach for computational tool (14) or hybrid multimodal imaging (34) has also broadened the clinical applicability of local hemodynamics. Nonetheless, there is no gold standard technique in identifying local hemodynamic parameters, and their definition and widely accepted consensus on clinical utilization still need to be determined in future studies. In view of physiological assessment in the cardiac catheterization laboratory, lesion-specific ischemia can be estimated by changes in the value of coronary physiological indices across the target lesion (i.e., ΔFFR), which are obtained by pressure-guide wire pullback measurement (35, 36). In our study, we aimed to compare the clinical implications of ΔFFR_CT_ with WSS, APS, and PG in prediction of ACS risk and demonstrated that ΔFFR_CT_ had comparable predictability for culprit lesions causing ACS with that of WSS, APS, and PG; and there was no significant benefit when WSS, APS, or PG was added into the risk prediction model with impaired FFR_CT_, high-risk plaque, and ΔFFR_CT_. Given that ΔFFR_CT_ can reflect the level of WSS, APS, or PG from the strong association among them, our finding postulates that the measurement of ΔFFR might provide equivalent prognostic information, which can be obtained by assessment of WSS, APS, or PG. Therefore, lesion-specific ΔFFR measurement through well-defined FFR pullback estimation in addition to vessel-specific FFR measurement for dichotomous decision-making for revascularization can better predict ACS risk, since it can depict local hemodynamic environments such as WSS, APS, or PG.

## Limitations

The current study has several limitations. First, the comparison between culprit lesions and non-culprit lesions was performed based on intra-patient analysis. Second, the number of the study population is relatively small to generalize the comparability among local hemodynamic parameters. Subsequent large-scale studies are needed to validate the current findings. Third, the study design is retrospective, and there may be selection bias on lesion progression or vulnerability. Fourth, the well-correlated relationship among local hemodynamic parameters is not a new finding given that those variables are mathematically associated with each other during the estimation process, and dependency among hemodynamic parameters might result in an insignificant increase in clinical value when they were used in the same prediction model. Nonetheless, we showed this relationship comprehensively using *in vivo* data, and we suggested a more accessible metric for estimation of local hemodynamic environment. Fifth, the blood rheology of an individual patient was not incorporated into the calculation of hemodynamic parameters. This might affect the accuracy of estimation of local hemodynamic environment because WSS can be generally estimated as the product of the blood dynamic viscosity and the gradient of the axial velocity of the vessel wall. Sixth, resting local hemodynamic parameters were not available for the current analysis, and future studies are needed to compare the prognostic implications between resting and hyperemic indices.

## Conclusions

Local hemodynamic parameters are significantly correlated with each other, and all indices have a prognostic role in prediction of ACS risk. ΔFFR_CT_ or ΔFFR, easily measurable indices in clinical practice, can reflect local hemodynamics including shear stress or plaque force acting on a plaque, and its use in clinical practice can optimize risk stratification for ACS.

## Data Availability Statement

The datasets presented in this article are not readily available because data cannot be shared publicly due to the privacy of individuals that participated in the study. The data will be shared on reasonable request to the corresponding author. Requests to access the datasets should be directed to Bon-Kwon Koo, bkkoo@snu.ac.kr.

## Ethics Statement

The studies involving human participants were reviewed and approved by Seoul National University Hospital. Written informed consent for participation was not required for this study in accordance with the national legislation and the institutional requirements.

## Author Contributions

SY and B-KK: conception, design, analysis, and interpretation of data, drafting and revising of manuscript, and final approval of the manuscript submitted. GC, JZ, JL, DH, J-HD, C-WN, E-SS, Y-SC, S-YC, EC, BN, KN, HO, MP, BB, TK, TA, and CT: interpretation of data, revising of manuscript, and final approval of the manuscript submitted. All authors contributed to the article and approved the submitted version.

## Funding

This study received funding from HeartFlow, Inc. The company performed the CFD analysis, but the funder was not involved in the study design, collection, analysis, interpretation of data, the writing of this article, or the decision to submit it for publication.

## Conflict of Interest

GC and CT are employees and shareholders of HeartFlow, Inc. BN has received institutional unrestricted research grants from Siemens and HeartFlow, Inc. BB has received institutional unrestricted research grants from Abbott, Boston Scientific, and Biotronik; has received consulting fees from Abbott, Opsens, and Boston Scientific; and is a shareholder for Siemens, GE, Bayer, Philips, HeartFlow, Inc., Edwards Life Sciences, Sanofi, and Omega Pharma. KN has received support from HeartFlow, Inc., Siemens Healthineers, and Bayer Healthcare. JL received a Research Grant from Abbott and Philips. J-HD received a Research Grant from Philips. B-KK received an Institutional Research Grant from Abbott, Philips, and HeartFlow, Inc. The remaining authors declare that the research was conducted in the absence of any commercial or financial relationships that could be construed as a potential conflict of interest.

## Publisher's Note

All claims expressed in this article are solely those of the authors and do not necessarily represent those of their affiliated organizations, or those of the publisher, the editors and the reviewers. Any product that may be evaluated in this article, or claim that may be made by its manufacturer, is not guaranteed or endorsed by the publisher.
