# Supplementary material for: Association Among Local Hemodynamic Parameters Derived From CT Angiography and Their Comparable Implications in Development of Acute Coronary Syndrome
Source: Front Cardiovasc Med. 2021 Sep 13;8:713835. doi: 10.3389/fcvm.2021.713835 (PMC8475759; doi:10.3389/fcvm.2021.713835)
Supplement: Supplementary file 1 [file Data_Sheet_1.docx]

**Supplementary Materials**

**Association Among Local Hemodynamic Parameters Derived from CT angiography and Their Comparable Implications in Development of Acute Coronary Syndrome**

**Supplementary Tables**

**Supplementary Figure Legends**

**Supplementary Tables**

**Supplementary Table 1. Correlation of local hemodynamic parameters with FFR_CT_.**

| Subgroups | Correlation between  FFR_CT_ and PG  (coefficient, r) | P-value | Correlation between  FFR_CT_ and WSS  (coefficient, r) | P-  value | Correlation between  FFR_CT_ and APS  (coefficient, r) | P-value |
| --- | --- | --- | --- | --- | --- | --- |
| **Vessel location** |  |  |  |  |  |  |
| LAD (n=81) | -0.502 | <0.001 | -0.249 | 0.020 | -0.033 | 0.765 |
| LCX (n=48) | -0.746 | <0.001 | -0.647 | <0.001 | -0.617 | <0.001 |
| RCA (n=81) | -0.372 | <0.001 | -0.211 | 0.058 | 0.052 | 0.642 |
| **Lesion location** |  |  |  |  |  |  |
| Proximal (n=98) | -0.711 | <0.001 | -0.564 | <0.001 | -0.097 | 0.343 |
| Mid (n=81) | -0.448 | <0.001 | -0.233 | 0.037 | -0.239 | 0.032 |
| Distal (n=37) | -0.430 | 0.008 | -0.253 | 0.130 | -0.220 | 0.192 |
| **% Diameter stenosis** |  |  |  |  |  |  |
| ≥50% (n=84) | -0.573 | <0.001 | -0.356 | <0.001 | -0.221 | 0.044 |
| <50% (n=132) | -0.298 | <0.001 | -0.195 | 0.025 | 0.178 | 0.042 |
| **High-risk plaque** |  |  |  |  |  |  |
| Yes (n=60) | -0.715 | <0.001 | -0.496 | <0.001 | -0.197 | 0.131 |
| No (n=156) | -0.383 | <0.001 | -0.248 | 0.002 | -0.047 | 0.564 |
| **FFR_CT_** |  |  |  |  |  |  |
| ≤0.80 (n=66) | -0.156 | 0.210 | 0.150 | 0.229 | -0.208 | 0.094 |
| >0.80 (n=150) | -0.601 | <0.001 | -0.625 | <0.001 | 0.125 | 0.128 |
| **Number of lesions in a vessel** |  |  |  |  |  |  |
| 1 (n=86) | -0.842 | <0.001 | -0.773 | <0.001 | -0.168 | 0.122 |
| ≥2 (n=130) | -0.431 | <0.001 | -0.202 | 0.021 | -0.079 | 0.370 |

FFR_CT_, coronary computedtomographic angiography-derived fractional flow reserve; LAD, left anterior descending artery; LAP, low-attenuation plaque; LCX, left circumflex artery; PG, pressure gradient; PR, positive remodeling; RCA, right coronary artery; WSS, wall shear stress.

**Supplementary Figure Legends**

**Supplementary Figure 1. Association of local hemodynamics with FFR_CT_.**

APS, axial plaque stress;FFR_CT_, coronary computedtomographic angiography-derived fractional flow reserve; WSS, wall shear stress.
