# Supplementary figures and images for: Association Among Local Hemodynamic Parameters Derived From CT Angiography and Their Comparable Implications in Development of Acute Coronary Syndrome
Source: Front Cardiovasc Med. 2021 Sep 13;8:713835. doi: 10.3389/fcvm.2021.713835 (PMC8475759; doi:10.3389/fcvm.2021.713835)

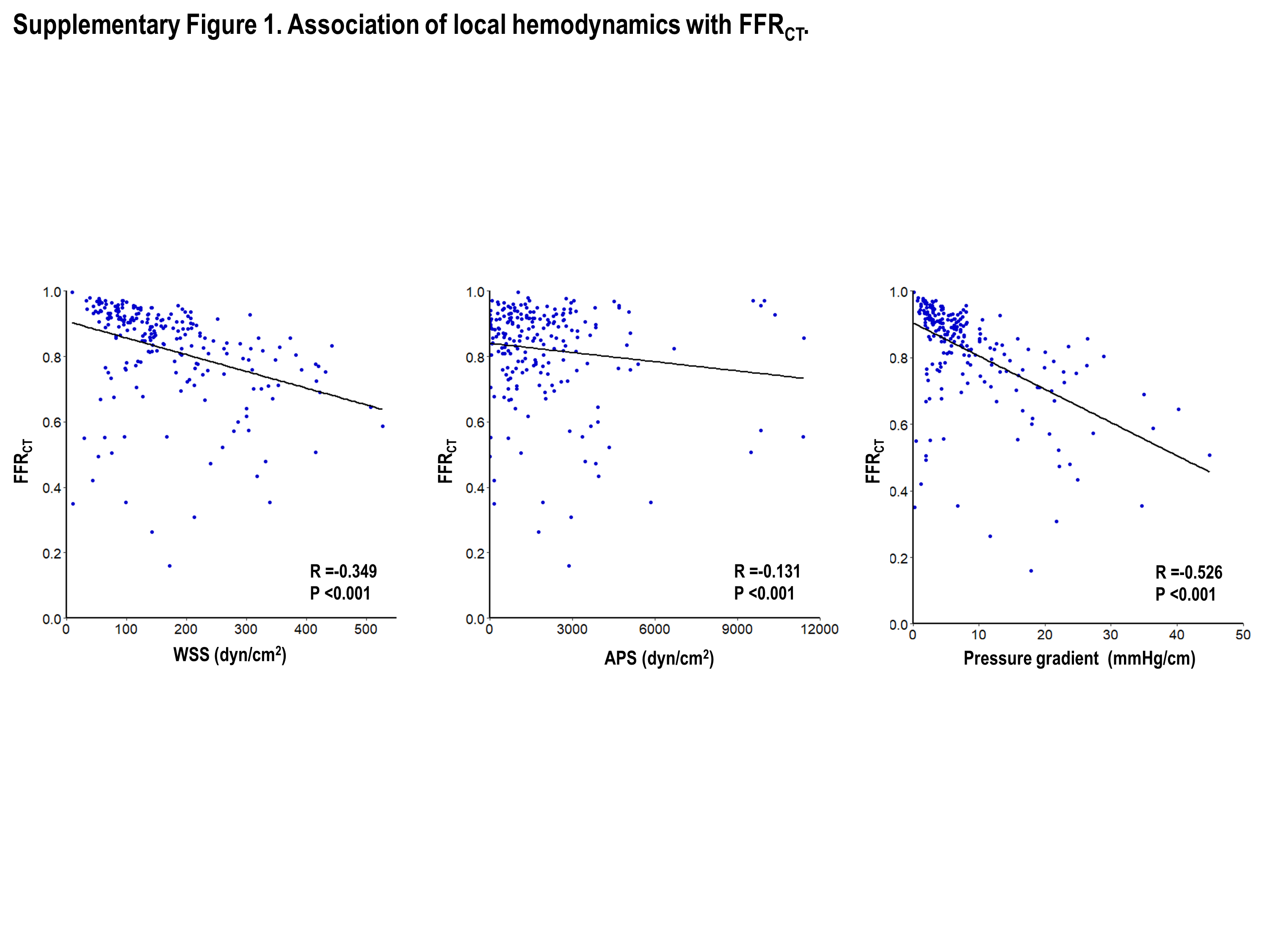

Supplement: Supplementary file 2 [file Image_1.TIF]
